# Understanding Medical Students’ Attitudes Toward Learning eHealth: Questionnaire Study

**DOI:** 10.2196/17030

**Published:** 2020-10-01

**Authors:** Kjeld Vossen, Jan-Joost Rethans, Sander M J van Kuijk, Cees P van der Vleuten, Pieter L Kubben

**Affiliations:** 1 Maastricht University Medical Center Maastricht Netherlands; 2 Skillslab Maastricht University Medical Center Maastricht Netherlands; 3 Department of Clinical Epidemiology and Medical Technology Assessment Maastricht University Medical Center Maastricht Netherlands; 4 Department of Educational Development and Research Maastricht University Medical Center Maastricht Netherlands; 5 Department of Neurosurgery Maastricht University Medical Center Maastricht Netherlands

**Keywords:** eHealth, student opinion, mHealth, medical education, students, medicine, curriculum, digital skills

## Abstract

**Background:**

Several publications on research into eHealth demonstrate promising results. Prior researchers indicated that the current generation of doctors is not trained to take advantage of eHealth in clinical practice. Therefore, training and education for everyone using eHealth are key factors to its successful implementation. We set out to review whether medical students feel prepared to take advantage of eHealth innovations in medicine.

**Objective:**

Our objective was to evaluate whether medical students desire a dedicated eHealth curriculum during their medical studies.

**Methods:**

A questionnaire assessing current education, the need for education about eHealth topics, and the didactical forms for teaching these topics was developed. Questionnaire items were scored on a scale from 1 (fully disagree with a topic) to 10 (fully agree with a topic). This questionnaire was distributed among 1468 medical students of Maastricht University in the Netherlands. R version 3.5.0 (The R Foundation) was used for all statistical procedures.

**Results:**

A total of 303 students out of 1468, representing a response rate of 20.64%, replied to our questionnaire. The aggregate statement “I feel prepared to take advantage of the technological developments within the medical field” was scored at a mean value of 4.8 out of 10. Mean scores regarding the need for education about eHealth topics ranged from 6.4 to 7.3. Medical students did not favor creating their own health apps or mobile apps; the mean score was 4.9 for this topic. The most popular didactical option, with a mean score 7.2, was to remotely follow a real-life patient under the supervision of a doctor.

**Conclusions:**

To the best of our knowledge, this is the largest evaluation of students’ opinions on eHealth training in a medical undergraduate curriculum. We found that medical students have positives attitudes toward incorporating eHealth into the medical curriculum.

## Introduction

According to the World Health Organization (WHO) definition, eHealth is the use of information and communications technology (ICT) to provide enhanced health services to communities [1]. eHealth services are defined as telehealth, electronic health records, mobile health, social media, and big data [1]. Several articles show promising results when eHealth is being used in medical fields [2-11]. For instance, it has been shown that eHealth interventions help to improve medication adherence [4], glycemic control in diabetes patients [6], and self-care among heart failure patients as well as improve the outcomes of cardiac rehabilitation among coronary heart disease patients [7,8] and improve mental health [9,10]. Teleconsultation by general practitioners has been proven as an alternative to face-to-face consultations in certain situations [11]. A review of 58 systematic reviews showed that overall eHealth provides beneficial results in a wide variety of medical applications [12]. These developments improve the quality of care or maintain the current standard and reduce health care costs [13-15]. These studies indicate that eHealth would allow for a change clinical practice for the better by using technology. However, this requires a workforce that is prepared to practice medicine in a way where eHealth is integrated into clinical practice.

Literature about educating medical students in the field of eHealth is scarce [16]. Due to this scarcity, we concluded that the implementation of eHealth education into the medical curriculum is limited. A recent assessment of medical curricula in Sweden showed that only one university had concrete plans about implementing eHealth into their medical curriculum [17]. Another trial in Australia showed that none of the universities had established an eHealth program [18]. In addition, the European Health Parliament found that current health professionals do not feel adequately trained in eHealth and found that formal eHealth training is lacking from an early stage in the training of medical professionals [19]. Universities and their executives are aware of the lack of formal eHealth education, but the medical curriculum is already crowded and priorities are given to other subjects [18].

Studies with composite student groups show that including eHealth courses in curricula increases knowledge and awareness about the topic [20,21]. There are several studies focusing solely on medical students where specific eHealth topics were tested; for example, app development or telehealth consultation skills [21-24]. These studies all show that a course, or even just one class, enhances the knowledge about specific topics and is appreciated by students. All this research assumes the top-down idea that eHealth education is important and necessary [16-27].

It may seem logical to incorporate eHealth into the medical curriculum; however, we do not know students’ perceptions about this. In fact, we could not find any articles that attempted to find out where students stand with regard to eHealth education. It might be that students are unaware of the lack of eHealth training and, therefore, do not feel the need for additional education. If students feel that education about eHealth is unnecessary, a different approach to teaching them is needed, compared to when students feel like they need more education about eHealth. The goal of our study is to evaluate whether medical students feel prepared to take advantage of eHealth innovation in medicine.

## Methods

### Setting

The following study was conducted at the medical school of Maastricht University in the Netherlands between February and May 2018. During the 6-year-long undergraduate medical curriculum—the duration of the bachelor and master programs are 3 years each—there is no formal education about eHealth. The most likely way students might encounter eHealth is through their medical rotations.

### Ethics Approval and Consent to Participate

This study was not submitted nor approved by an institutional ethics committee because we did not deem this necessary in accordance with Dutch law. Dutch ethical law states that ethical approval is only necessary in the case of medical research including human test subjects, as can be read in the Medical Research Involving Human Subjects Act (*Wet medisch-wetenschappelijk onderzoek met mensen*, in Dutch), paragraph 2 [28]. Our research was aimed at the improvement of education and training without submitting the participants to any medical intervention. Therefore, this was not deemed medical research but educational research. Consent to participate was obtained at the beginning of the questionnaire administration. When the participants opened the questionnaire online, they were met with a statement stating that participants consented to participate and the data could be used for research purposes.

### Questionnaire

#### Overview

We were unable to find any pre-existing questionnaire that assessed students’ attitudes toward learning eHealth; therefore, we decided to create our own questionnaire. To assess students’ attitudes toward learning eHealth, we developed a Dutch questionnaire using Google Forms that was made accessible for the participants to fill out from February 2018 until May 2018. We chose this type of survey in order to reach as many students as possible and to increase the number of responses. Most of the students spend little time at the university and prefer to either work from home or spend their time learning at the hospital during their clinical rotations. The most effective way for us to reach these students was by using a format such as Google Docs.

We based the statements on the WHO definition of eHealth. We incorporated a question about every aspect of this definition in our questionnaire. Furthermore, we specified between the bachelor and master curricula to see if there were any significant differences between the two subgroups.  Because there is currently no dedicated eHealth education course offered, we could not evaluate these topics; we could only assess whether or not our students would want access to education about the topics.

Excluding the personal questions, the questionnaire contained 18 or 20 statements, depending on the participants’ study phase: bachelor or master program. The statements, which were translated into English for this paper, are listed in the Results section later in this article. The statements about current education and didactical options were piloted among 6 master students and 4 bachelor students, who found the statements clear and comprehensive. The master students’ questionnaire contained two additional statements about the medical rotations and in-class education during the master program. The rest of the questionnaire was identical for both master and bachelor program students.

#### Characteristics

The first section of the questionnaire gathered participant characteristics (see Table 1). The answer given for the question *study level* routed participants to the next section. Bachelor students were routed to a form evaluating solely the bachelor education. Master students were routed to a form evaluating both the bachelor and master education. Participants could indicate how long ago they finished their medical education, choosing between *longer than 2 years ago* or *between 0 and 2 years ago*.

**Table 1:** Participant characteristics.

Characteristic		Value (N=303), n (%)
**Gender**		
	Female	215 (71.0)
	Male	88 (29.0)
**Age (years)**		
	<20	38 (12.5)
	20-25	226 (74.6)
	>25	39 (12.9)
**Technical skill level**		
	User	257 (84.8)
	Advanced	43 (14.2)
	Expert	3 (1.0)
**Study level**		
	Bachelor	120 (39.6)
	Master	183 (60.4)

#### Current Education

The second section reviewed current education. All questions about the current education and didactical options were answered using a 10-point Likert scale, ranging from 1 (fully disagree) to 10 (fully agree). We decided on a scale from 1 to 10 because our students are used to be graded using this scale, where a grade above 5.5 is considered satisfactory. Both the bachelor and master students’ forms contained the following statement: “I feel prepared to take advantage of the technological developments within the medical field.” This statement was included as an aggregate global judgment about the entire curriculum.

#### eHealth Topics and Didactical Format

After the section about the students’ current education, the questionnaire was the same for every participant. The third section evaluated how students felt about different eHealth subjects in the medical curriculum. Topics listed were chosen based on the WHO definition of eHealth, namely, mobile apps, telemonitoring, applying modern technology in practice, data science, and machine learning. The last seven statements evaluated which didactical format the students preferred.

At the end of the survey, students had the option to give feedback or add explanations to their answers. It was not possible to skip questions or statements during the questionnaire; therefore, all questionnaires we received were complete.

### Participants

A total of 316 medical students enroll at Maastricht University’s medical school every year, resulting in a total of about 1896 students. We promoted the questionnaire via social media groups that are only accessible by our university medical students. The local medical student association allowed us to use their newsletter to promote the questionnaire. In addition to this, we reached out to medical students through their social media accounts by sending them a personal message about the questionnaire with a link to the Google Form. There was no incentive for students to fill out the form and there were no negative consequences if students did not fill out the form. The inclusion criterion was as follows: medical student actively studying at the time of the survey. The reason for applying this criterion was that all participants will have studied the same curriculum.

### Statistical Analysis

Descriptive statistics consisting of the mean, standard deviation, 95% confidence interval, and Cronbach α were calculated, and box and whisker plots were drawn to give a graphic, representation of the results. R version 3.5.0 (The R Foundation) was used for all statistical procedures.

## Results

### Demographics

In total, 1468 invitations were sent to medical students to participate in the survey using social media, WhatsApp, and the platforms provided by the medical student association. There were 303 responses to the questionnaire, giving a response rate of 20.64% (303/1468). Characteristics of the participants are listed in Table 1. Most participants were female (215/303, 71.0%). The mean age was 22 years (range 20-25). This is comparable to the average age of medical students at our university. Master students were the largest subgroup, with a total of 183 participants out of 303 (60.4%). The other 120 participants were bachelor students (39.6%).

### Results From the Survey

**Figure 1:**
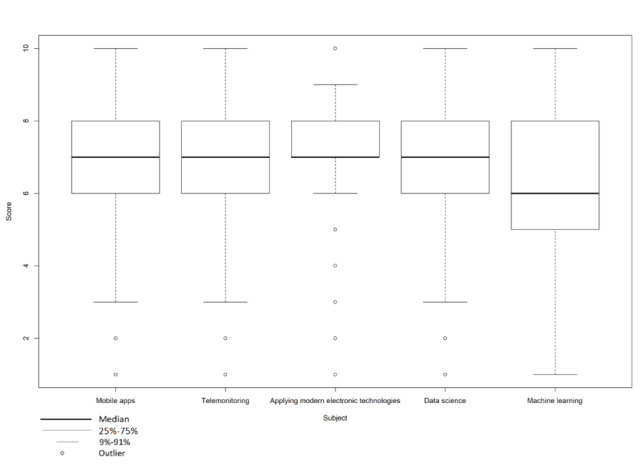
Students' attitudes toward given topics in the medical curriculum (N=303). Scores range from 1 (fully disagree) to 10 (fully agree).

Table 2 shows how prepared the students feel to use eHealth in their future medical practice. The global aggregate statement “I feel prepared to take advantage of the technological developments within the medical field” scored a low value of 4.8 out of 10 (95% CI 4.6-5.0). Figure 1 shows the students’ attitudes toward different topics upon which we questioned them. Students assigned positive values (ie, a score of 6 or higher) to all topics, meaning that they would like to receive more education about a given topic. The least popular topic was that of machine learning, which had a mean score of 6.4 (SD 1.8, 95% CI 6.2-6.9). The most popular topic was that of applying modern electronic technologies in health care, which had a mean score of 7.3 (SD 1.6, 95% CI 7.1-7.4). When comparing the results from the statements about the current curriculum with results from statements about the eHealth topics, there was a difference between the two. There was a discrepancy between eHealth-related content in current medical education and the amount of eHealth training that is considered useful by medical students.

**Table 2:** Responses to statements regarding how prepared medical students feel with regard to eHealth and the education they would like to receive.

Category		Questionnaire statement	Score				
			Mean (SD)		95% CI		
**Type of student**							
	**Bachelor student (n=120)**						
		During the Bachelor of Medicine, there is enough education about the technological developments in medicine and eHealth.	4.9 (1.6)		4.7-5.1		
		I feel prepared to take advantage of the technological developments within the medical field.	4.8 (1.6)		4.6-5.0		
	**Master student (n=183)**						
		During the Bachelor of Medicine, there is enough education about the technological developments in medicine and eHealth.	4.3 (1.8)		4.1-4.8		
		During the Master of Medicine, there is enough education about the technological developments in medicine and eHealth.	4.6 (1.8)		4.4-4.8		
		During my medical rotations, I increase my experience with eHealth and the use of technology within health care.	5.1 (1.8)		4.9-5.3		
		I feel prepared to take advantage of the technological developments within the medical field.	4.8 (1.7)		4.6-5.0		
**Educational topics and didactical work format (N=303)**							
	**Educational topics**						
		During medical education, there should be more education about the use of mobile apps to support the treatment of a patient.	6.6 (1.7)		6.4-6.8		
		During medical education, there should be more education about the use of telemonitoring of patients.	6.7 (1.7)		6.5-6.9		
		During medical education, there should be more education about applying modern electronic technologies in health care.	7.3 (1.5)		7.1-7.4		
		During medical education, there should be more education about using data science in medicine.	6.9 (1.8)		6.7-7.1		
		During medical education, there should be more education about machine learning in medicine.	6.4 (1.9)		6.2-6.9		
	**Didactical work format**						
		I would like to receive education about technological developments in the form of lectures.	5.5 (2.1)		5.2-5.7		
		I would like to receive education about technological developments in the form of tutorials.	6.7 (1.8)		6.5-6.9		
		I would like to receive education about technological developments in the form of real-life scenarios and case descriptions.	6.8 (2.2)		6.6-7.1		
		I would like to receive education about technological developments in the form of developing my own health app or mobile app.	4.9 (2.7)		4.6-5.2		
		I would like to receive education about technological developments in the form of video lectures.	5.1 (2.3)		4.8-5.3		
		I would like to receive education about technological developments in the form of short video material.	5.9 (2.2)		5.6-6.1		
		I would like to receive education about technological developments in the form of remotely following a real-life patient under the supervision of a doctor.	7.2 (2.0)		7.0-7.5		

**Figure 2:**
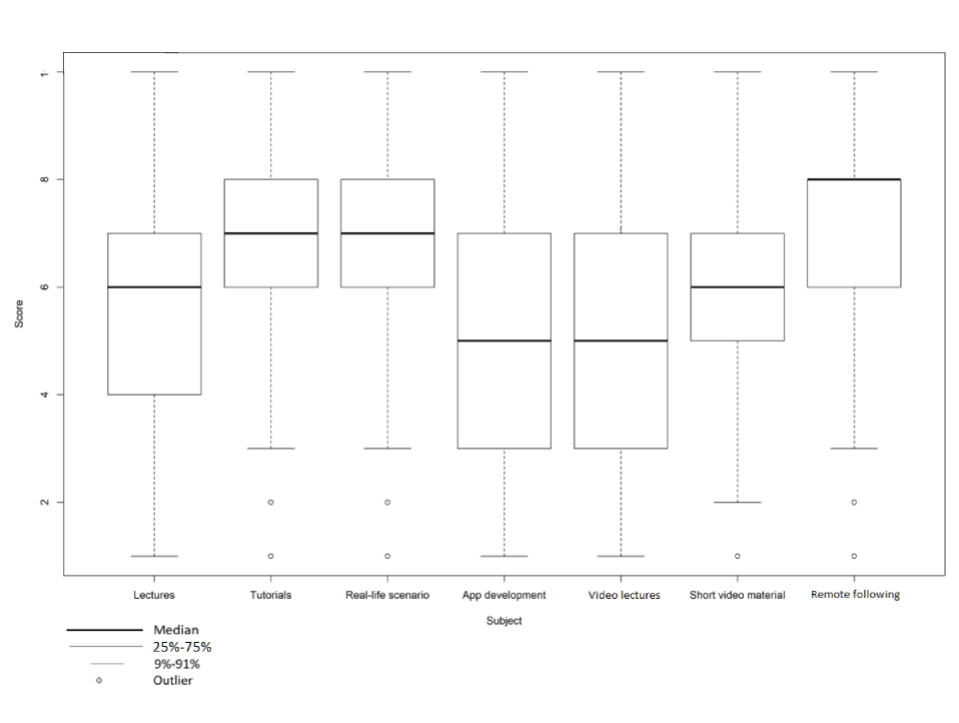
Students' opinions about how they would like to receive education about eHealth topics (N=303). Scores range from 1 (fully disagree) to 10 (fully agree).

Figure 2 lists the didactical format the students would prefer when learning about the given topics. The most popular format, with a mean score of 7.2 (SD 2.0), was to remotely follow a real patient under the supervision of a doctor. The least popular form of education was the development of a student’s own health app or mobile app. This only received a mean score of 4.9 (SD 2.7).

## Discussion

### Principal Findings

To the best of our knowledge, this is the largest evaluation of students’ opinions on eHealth training in a medical undergraduate curriculum. We found that medical students have positive attitudes toward incorporating eHealth into the medical curriculum. This study showed that students do not feel well prepared to take advantage of eHealth in medical practice. The students scored an average of 4.8 out of a maximum of 10 when asked how prepared they felt to take advantage of eHealth in medical practice, while the need for education about the given topics scored a minimum of 6.4 and a maximum of 7.3. These results might provide a basis from which to continue a discussion regarding integrating eHealth into the medical curriculum.

eHealth has been proven to be effective in clinical practice [1-13,29,30]; however, eHealth has not yet been implemented into the working standards of many doctors [31-33]. Prior research indicates that training and education for all those involved with implementation and the use of eHealth is a key factor for the successful incorporation of eHealth [13-16,30]. There are various obstacles to implementing eHealth into clinical practice. The two biggest barriers to the use of eHealth in clinical practice are as follows: *technically challenged staff* (11%) and *resistance to change* (8%) [32].

Resistance to change is linked to several factors; lack of knowledge and skill obsolescence are major contributing factors [34]. Lack of eHealth skills and training is prevalent among the current medical workforce. This has been shown to be a major barrier to eHealth adoption [35-38]. This results from the fact that the workforce is not adequately trained to implement eHealth in medical practice [25-27,39]. Awareness and knowledge of what needs to change are essential in enabling change [40]. It has previously been shown that education can be used to overcome resistance to change [41,42]. Therefore, education about eHealth for those involved, in this case the undergraduate students, can lessen the barriers previously mentioned and help to create a workforce that is open and able to use eHealth in their daily practice. Many universities do not have dedicated eHealth training in the current curriculum, adding to the resistance to change [17,18]. Universities and their executives are aware that eHealth training is important, but due to the already overcrowded curricula with competing interests, implementation is lacking [18]. Our results suggest that we should dedicate more time to eHealth training, even though the curriculum is already overcrowded. This overcrowding can be overcome by changing education in the same way that clinical practice is being changed by eHealth. Case-based discussions that are based on a fictional patient can be replaced by cased-based discussions that are based on a real-life patient being remotely monitored in in a hospital, simulation patients can be replaced by teleconference simulation patients, and clinical rotations can incorporate remote care. eHealth education can be combined with the current subjects taught in the curriculum without taking up more time. By thinking of eHealth education the same way as eHealth implementation into clinical practice, it should be possible to incorporate this education into the medical curriculum. eHealth is not some added technology that takes up time; it should be an integrated aspect in clinical practice that improves the quality of care while reducing the workload. Showing students early on during their education that eHealth can be used in these ways might add to a certain digital mindset that is needed to use eHealth to its full extent as a clinician.

Medical students are not receiving enough education to prepare them for eHealth competency. The current workforce is not adequately trained to implement new technologies in working practice [17,18,25,26,34-39,43]. The data we gathered support this because the participants indicated they do not feel prepared to benefit from the technological developments in health care. This holds true for both the bachelor and master students. The master students gave the amount of eHealth training in the bachelor program a lower score, which might indicate that during their medical rotations they were confronted by the fact that their digital skills were lacking. The students indicated that during their rotations, they were not learning enough about eHealth and the use of technology within health care. This might be due to the fact that there is no mention of eHealth skills in the national Dutch framework, which states what competencies a future doctor needs. During these medical rotations, opportunities to develop digital skills are lacking. Their teachers make up the current workforce of doctors. We previously established that this workforce is not adequately prepared to take advantage of the possibilities offered by eHealth. Therefore, we cannot expect them to train the new generation of doctors to attain sufficient digital skills. It would be possible to integrate digital skills education during the medical rotations if taught by eHealth or ICT professionals. The skills could then be further developed during medical rotations, providing a solid foundation for the future workforce.

If eHealth education is implemented early in medical education, this might result in professionals being able to benefit from eHealth. Both bachelor and master students indicated that they feel a lack of this type of education. We concluded from this that it would be beneficial to start training the students during their bachelor phase. This would mean that they would be better prepared to use the skills they have learned during their medical rotations and, therefore, gain practical experience using their skills as soon as possible.

This study works as a basis to support the need for eHealth education among medical students. There is still a lot of work that needs to be done with regard to a framework that defines which eHealth competencies are needed by future doctors, what eHealth subjects should be prioritized, and how students should be taught these subjects.

Another factor that may cause insufficient attention with regard to eHealth training is the assumption that today’s students are up to date with technological developments, including an understanding of eHealth, because of the widespread use of technology among this generation [20,44]. However, prior research demonstrates that undergraduate students do not have this knowledge [25-27]. It could mean that health care is missing out on some of the potential benefits of eHealth due to this assumption. Nevertheless, it is important to use the skills that students have already gathered in the digital age while training them for their professions as future doctors. We should, therefore, always invite student panels while creating a future eHealth curriculum.

Incorporating formal eHealth education into the medical curriculum may contribute to creating a necessary digital mindset [27,45]. This digital mindset means more than just the use of tools [31,32]; it would mean that medical professionals would start to think differently regarding how to provide health care. An example would be to think about how to change from traditional in-hospital care to future health care within patients’ homes. We are increasingly able to gather large amounts of patient data and need medical staff that can think in creative ways in using this data [43]. We hope that by supporting the development of a digital mindset in future medical staff that they will see opportunities in techniques such as data science, machine learning, and deep learning.

### Strengths and Limitations

The first limitation in this study is a low response rate amounting to 20.64% (303/1468). We used Google Forms to poll our students. This platform provides an easy and accessible way for participants to fill out forms. The downside was that it was impossible to make sure that people did not fill out the form multiple times. However, it seems unlikely that people filled out multiple forms. If duplicates had been filled out by certain individuals, this would change the demography of our sample. However, the demography of the participants matches the demography of our student population. Besides that, we checked the time stamps of all the forms to check if there were identical forms filled out in short succession. After checking time stamps and responses of each questionnaire, we found that there was no evidence that duplicate forms had been filled out.

Before we created the questionnaire, we looked for similar surveys, but we were not able to find any related to medical education. That is why we compiled our own questionnaire using the WHO definition for eHealth and recent literature. We took a pragmatic approach when creating our questionnaire, and the final questionnaire was not validated. All participants were from a single university in the Netherlands. This may limit the external validity of our results. During our research, we have noticed that there is a limited amount of literature about eHealth training for medical students and, therefore, we assume that most universities do not have a dedicated eHealth program. In this case, our results could inform other universities about the lack of eHealth training in their curriculum.

### What This Paper Adds

The previous statements saying that eHealth education is necessary were top-down statements. This paper adds the students’ views on eHealth and shows that students feel that they are not prepared to take advantage of the possibilities provided by eHealth. This paper shows that students want more education about eHealth topics.

### Conclusions

This study demonstrates that students consider themselves insufficiently prepared for the digital aspect of their future medical practices and that they support greater attention to eHealth in the medical curriculum. This study indicates that the lack of eHealth education is not something that is experienced only by researchers who write about eHealth education but also by the medical students themselves.

## Abbreviations

ICT: information and communications technology
WHO: World Health Organization
